# Allogeneic hematopoietic stem cell transplantation in ERCC6L2 disease^∗^

**DOI:** 10.1182/bloodadvances.2025018349

**Published:** 2026-02-04

**Authors:** Marja Hakkarainen, Flore Sicre de Fontbrune, Ilse Kaaja, Suvi Douglas, Jean-Hugues Dalle, Antonio Maria Risitano, Austin Kulasekararaj, Abdulrahman Alsultan, Corey Cutler, Vincent T. Ho, Eva Hellström-Lindberg, Stephan Mielke, Anders E. Myhre, Rawad Rihani, Mayada Abu Shanap, Hasan Hashem, Akiko Shimamura, R. Grant Rowe, Franziska Auer, Fabian Beier, Lana Desnica, Rachael Hough, Syed Rafat Ali Jafri, Mouhab Ayas, Laura Jardine, Eugenia Fernandez Mellid, Irene Corrales, Deborah Richardson, Namik Yasar Özbek, Anna Zaniewska-Tekieli, Jolanta Gozdzik, Samppa Ryhänen, Riitta Niinimäki, Kirsi Jahnukainen, Urpu Salmenniemi, Outi Kilpivaara, Ulla Wartiovaara-Kautto

**Affiliations:** 1 Applied Tumor Genomics and Research Program, Research Programs Unit, University of Helsinki, Helsinki, Finland; 2 Department of Hematology, Helsinki University Hospital Comprehensive Cancer Center and University of Helsinki, Helsinki, Finland; 3 Department of Medical and Clinical Genetics/Medicum, Faculty of Medicine, University of Helsinki, Helsinki, Finland; 4 Hematology Transplant Unit, National French Reference Center for Aplastic Anemia and Paroxysmal Nocturnal Hemoglobinuria, Centre de Référence des Aplasies Médullaires Acquises et Constitutionnelles, Hôpital Saint-Louis, Paris, France; 5 Pediatric Immunology and Hematology Department and Centre de Référence des Maladies Rares Aplasies Médullaires, Robert Debré Hospital, Groupe Hospitalier Universitaire Assistance Publique-Hôpitaux de Paris Nord Université de Paris Cité, Paris, France; 6 Hematology, Hospital “San Giuseppe Moscati,” Avellino, Italy; 7 Severe Aplastic Anemia Working Party, European Bone Marrow Transplant, Leiden, The Netherlands; 8 Department of Haematological Medicine, King’s College Hospital NHS Foundation Trust, London, United Kingdom; 9 Oncology Center, King Saud University Medical City, Riyadh, Saudi Arabia; 10 Department of Pediatrics, College of Medicine, King Saud University, Riyadh, Saudi Arabia; 11 Division of Transplantation and Cellular Therapy, Department of Medical Oncology, Dana-Farber Cancer Institute, Boston, MA; 12 Center for Hematology and Regenerative Medicine, Department of Medicine, Karolinska Institutet, Stockholm, Sweden; 13 Department of Haematology, Oslo University Hospital, Oslo, Norway; 14 Department of Pediatric Hematology/Oncology and Bone Marrow Transplantation, King Hussein Cancer Center, Amman, Jordan; 15 Dana Farber/Boston Children’s Cancer and Blood Disorders Center, Harvard Medical School, Boston, MA; 16 Department of Pediatrics, Technical University of Munich, Munich, Germany; 17 Department of Hematology, Oncology, Hemostaseology and Stem Cell Transplantation, Medical Faculty, Rheinisch-Westfälische Technische Hochschule Aachen University, Aachen, Germany; 18 Center for Integrated Oncology Aachen Bonn Cologne Düsseldorf, Aachen, Germany; 19 Division of Hematology, Department of Internal Medicine, University Hospital Centre Zagreb, Zagreb, Croatia; 20 Children and Young People’s Cancer Service, University College London Hospitals NHS Foundation Trust, London, United Kingdom; 21 Section of Stem Cell Transplant & Car T Cell Therapy, Department of Pediatrics Hematology & Oncology, King Faisal Specialist Hospital & Research Center, Riyadh, Saudi Arabia; 22 Northern Centre for Cancer Care, Newcastle upon Tyne Hospitals NHS Foundation Trust, Newcastle upon Tyne, United Kingdom; 23 Biosciences Institute, Newcastle University, Newcastle upon Tyne, United Kingdom; 24 Complexo Hospitalario Universitario de Santiago de Compostela, A Coruña, Spain; 25 Laboratori de Coagulopaties Congènites, Banc de Sang i Teixits, Barcelona, Spain; 26 Medicina Transfusional, Vall d’Hebron Institut de Recerca, Universitat Autònoma de Barcelona, Barcelona, Spain; 27 Centro de Investigación Biomédica en Red de Enfermedades Cardiovasculares, Instituto de Salud Carlos III, Madrid, Spain; 28 Department of Haematology, University Hospital Southampton, Southampton, United Kingdom; 29 Department of Pediatric Hematology and Oncology, Ankara City Hospital, University of Health Sciences, Turkey University of Health Sciences, Ankara, Turkey; 30 Department of Transplantation, University Children’s Hospital in Krakow, Krakow, Poland; 31 Department of Clinical Immunology and Transplantation, Medical College, Jagiellonian University, Transplantation Center, University Children’s Hospital in Krakow, Krakow, Poland; 32 Division of Hematology, Oncology, and Stem Cell Transplantation, Children’s Hospital, and Pediatric Research Center, University of Helsinki and Helsinki University Hospital, Helsinki, Finland; 33 Department of Pediatrics, Oulu University Hospital and PEDEGO Research Unit, University of Oulu, Oulu, Finland; 34 Department of Women’s and Children’s Health, Nordic Center for Fertility Preservation Research Lab Stockholm, Karolinska Institutet and University Hospital, Stockholm, Sweden; 35 iCAN Digital Precision Cancer Medicine Flagship, University of Helsinki, Helsinki, Finland; 36 K. Albin Johansson Cancer Researcher Fellow, Foundation for the Finnish Cancer Institute, Helsinki, Finland

## Abstract

The hallmark of ERCC6L2 disease (ED) is a highly penetrant progression from bone marrow failure to erythroid-predominant, *TP53*-mutated myeloid malignancy with a dismal prognosis. Allogeneic hematopoietic stem cell transplant (HSCT) remains the only potentially curative option, but concerns exist regarding transplant-related toxicities (TRT) due to the underlying DNA repair defect. To the best of our knowledge, this is the first study to report a systematic analysis of HSCT in ED. We conducted a retrospective multicenter study involving 45 patients with ED who underwent HSCT in 2004-2024. The primary outcomes were overall survival (OS), TRT, and nonrelapse mortality (NRM). The 1-year and 3-year OS were 79% (95% confidence interval [CI], 66-91) and 54% (95% CI, 35-73), respectively. Previous history of excess blasts significantly predicted inferior survival (hazard ratio [HR], 6.8; 95% CI, 2.2-20.3; *P* < .001), with a median survival of 12 months (95% CI, 0-24). Grade 3 to 5 endothelial toxicities occurred in 27% of patients and were associated with higher NRM (HR, 7.7; 95% CI, 1.5-38.8; *P* = .016). The use of nontreosulfan-based myeloablative conditioning (MAC) regimens increased the risk of endothelial complications compared with reduced-intensity conditioning (RIC; HR, 4.9; 95% CI, 1.1-22.0; *P* = .040), whereas outcomes with treosulfan-based MAC were comparable to RIC. In summary, allogeneic HSCT is a viable curative strategy for ED when performed before transformation to an aggressive malignancy, for example myelodysplasia with excess blasts or acute myeloid leukemia. However, the elevated incidence of endothelial toxicity highlights the importance of optimizing conditioning intensity and enhancing peritransplant monitoring in this population.

## Key Points

• Allogeneic HSCT is a curative option for ED if performed before progression to aggressive malignancy.
• Patients with ED have a significant risk of developing severe endothelial damage events after HSCT.

## Visual Abstract

**Figure:**
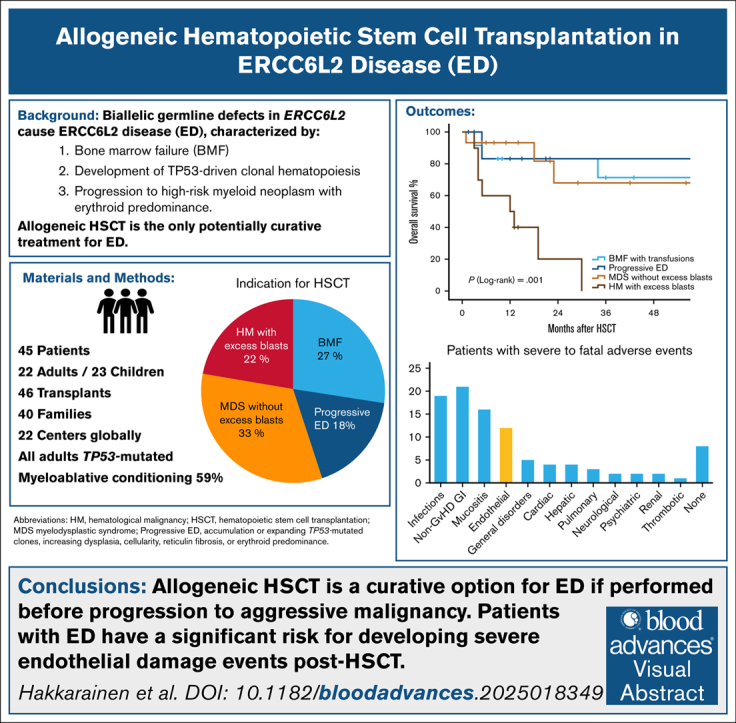

## Introduction

Biallelic germ line defects in *ERCC6L2* result in ERCC6L2 disease (ED), characterized by bone marrow failure (BMF), development of exclusively *TP53* mutation-driven clonal hematopoiesis, and further progression to a high-risk myeloid neoplasm with erythroid predominance.^1^, ^2^, ^3^, ^4^, ^5^, ^6^, ^7^, ^8^ The caveats for disease acceleration in bone marrow (BM) are increasing dysplasia, cellularity, erythroid predominance, reticulin fibrosis, and expansion or accumulation of *TP53*-mutated clones.^8^ Currently, allogeneic hematopoietic stem cell transplant (HSCT) is the only potentially curative therapy for ED. Our preliminary data suggested that patients with BMF at the time of HSCT have superior overall survival (OS) compared with patients who underwent transplant due to hematological malignancies (HMs).^8^ The underlying disease biology, particularly the role of ERCC6L2 in DNA repair, raises concerns for patients undergoing HSCT.^4^^,^^9^, ^10^, ^11^ Other inherited BMF syndromes (IBMFSs) have been linked to increased risks of transplant-related toxicity (TRT) and nonrelapse mortality (NRM).^12^, ^13^, ^14^ Furthermore, *ERCC6L2* variants have been associated with increased sensitivity to ionizing radiation, radiosensitivity, and abnormalities in immunoglobulin class-switch recombination.^2^^,^^10^^,^^11^^,^^15^ Given the lack of successful therapies for *TP53* mutation-driven hematological HMs, it is critical to explore and optimize HSCT outcomes in patients with ED.

In this study, we aim to conduct a thorough investigation of HSCT in ED by analyzing transplant characteristics, TRTs, and patient outcomes. Our objectives are to establish a knowledge base for determining the optimal timing of HSCT, and identify the most suitable conditioning regimen tailored to each patient’s needs.

## Methods

The study was approved by the Helsinki University Hospital Ethics Committee (protocols 206/13/03/03/2016 [amendment 1/2023] and 303/13/03/01/2011), the Severe Aplastic Anemia Working Party of the European Bone Marrow Transplant (EBMT), and the local institutional review boards for centers outside the EBMT Registry. Written informed consent was obtained from all patients or their legal guardians. The study included individuals with a confirmed genetic diagnosis of biallelic germ line variants in *ERCC6L2* undergoing HSCT (N = 45). Subjects were identified from previously published studies^8^^,^^16^, ^17^, ^18^ and personal contacts (n = 37), and through a survey sent to all EBMT centers by the Severe Aplastic Anemia Working Party (n = 8). Participating centers (n = 22) conducted a retrospective chart review using pseudonymized patient data from medical records. In addition, we used a detailed questionnaire to collect data on TRTs. All *ERCC6L2* variants were originally classified as disease-causing at the referring institutions. We reclassified the variants according to the American College of Medical Genetics and Genomics, and the Association for Molecular Pathology guidelines^19^ (supplemental Table 1). We applied Rare Exome Variant Ensemble Learner scores^20^ or, when unavailable, Mutation Prediction 2 scores^21^ for in silico pathogenicity assessment of missense variants (Association for Molecular Pathology criterion PP3, supporting evidence for pathogenicity based on computational predictions).^22^ Splicing probabilities were evaluated using VarSome Germline Classification (version 13.13.1).

### Definitions

Patients with aplastic anemia were classified as having BMF. Patients exhibiting an accumulation or expanding BM *TP53-*mutated clones, increasing dysplasia, cellularity, reticulin fibrosis, or erythroid predominance were categorized as having progressive ED. Diagnosis of myelodysplastic syndrome (MDS) and acute myeloid leukemia (AML) followed the latest International Consensus Classification of Myeloid Neoplasms and Acute Leukemias.^23^ We considered treosulfan at 14 g/m^2^ for 3 consecutive days as a myeloablative conditioning (MAC) regimen. Engraftment was defined as a sustained absolute neutrophil count of ≥0.5 × 10^9^/L for 3 consecutive days without administration of growth factors.^24^ Acute and chronic graft-versus-host disease (aGVHD and cGVHD) were assessed and graded based on established published criteria in each study center.^25^^,^^26^ NRM was considered as death not due to underlying disease. TRTs were graded according to Common Terminology Criteria for Adverse Events 5.0. The criteria for endothelial complications followed the EBMT classifications, and included capillary leak syndrome, cytokine release syndrome, engraftment syndrome (ES), periengraftment respiratory distress syndrome (PERDS), transplant-associated thrombotic microangiopathy (TA-TMA), and sinusoidal obstruction syndrome (SOS), previously known as hepatic veno-occlusive disease.^27^, ^28^, ^29^

### Statistical methods

We evaluated the differences in continuous variables using the Mann-Whitney U test, and in categorical variables using the Fisher exact and χ^2^ tests; all tests were 2-tailed. Categorical variables are presented as percentages within the subset with available data. We studied disease-free survival (DFS), event-free survival (EFS), and OS probabilities by calculating Kaplan-Meier curves, and compared them using the log-rank test. For the DFS analysis, we included only morphological relapses, defined by morphological and clinical evidence of disease activity. For EFS, we considered graft failure, morphological relapse, and death from any cause as events. We used Cox proportional hazard models for hazard ratios (HR) in univariable and multivariable analyses. Follow-up time started at the date of HSCT and ended at death, and surviving patients were censored at the last follow-up. Cumulative incidences (CIn) of aGVHD, cGVHD, relapse, and NRM were analyzed separately in a competing risks framework. We used IBM SPSS Statistics (version 29.0, Armon, NY) and R version 3.6.0 (Development Core Team, Vienna, Austria) for statistical analyses.

## Results

### Characteristics of study patients

**Figure 1.**
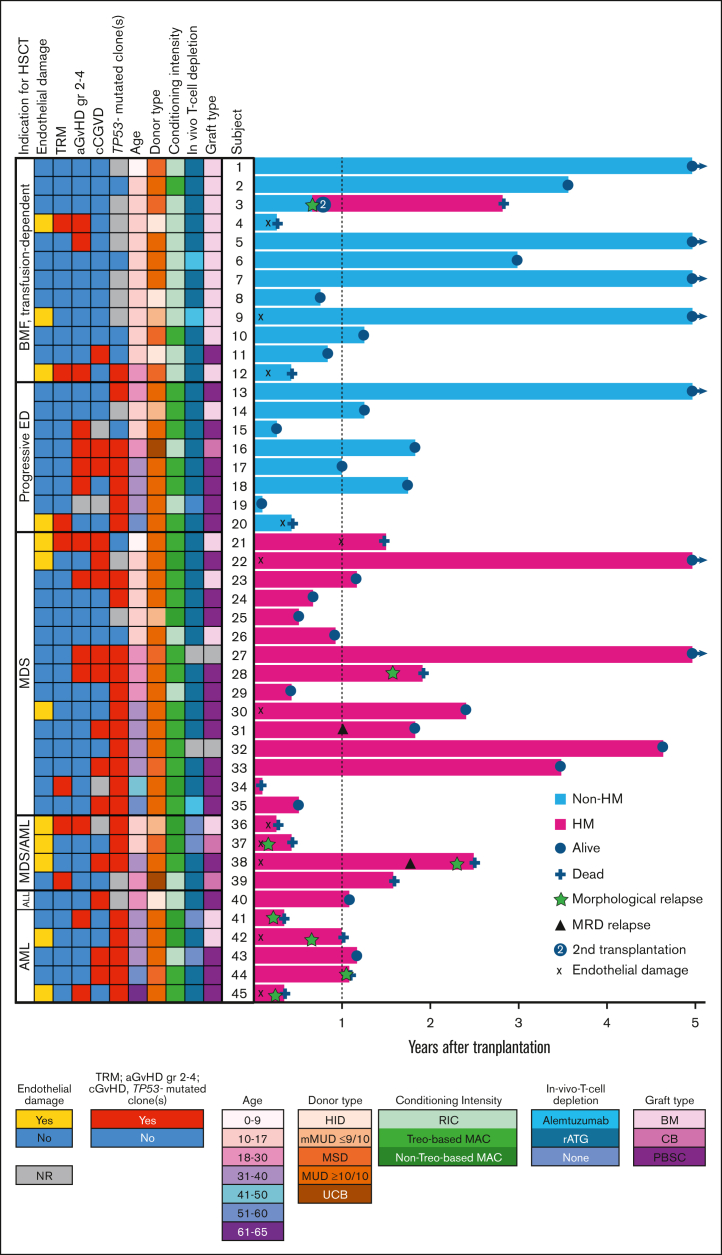
**Combined oncoplot and swimmer plot of study subjects.** The left panel (oncoplot) summarizes key clinical events and characteristics of each patient. The right panel (swimmer plot) illustrates individual patient timelines starting from the day of HSCT. Arrows indicate ongoing follow-up at the time of data cutoff. CB, cord blood; HID, haploidentical donor; mMUD, mismatched unrelated donor; MSD, matched sibling donor; MUD, matched unrelated donor; PBSC, peripheral blood stem cells; rATG, rabbit antithymocyte globulin; TRM, tranplant-related mortality; UCB, umbilical cord blood.

We explored details of allogeneic HSCTs (N = 46) in 45 patients with ED from 40 families (Table 1; Figure 1). Individual data, including pre-HSCT treatments and responses, are presented in supplemental Table 1. Study subjects aged <18 years (n = 22) were classified as pediatric. Transplants were performed during 200 to 2024, with the median year of 2021. We evaluated the effect of the transplant eras (2004-2015, n = 7; 2016-2020, n = 12; 2021-2024, n = 26), and found no statistically significant differences in baseline characteristics or OS between transplant eras (supplemental Table 2). Indications for HSCT were transfusion-dependent BMF (n = 12), proactive therapy for progressive ED (n = 8), and high-risk HMs (n = 25; detailed in Table 1). Eleven patients (5 with BMF and transfusion dependency, and 6 with HM) underwent HSCT before the diagnosis of ED was known, but were later found to have the condition through participation in a study involving germ line analysis. All adult patients with known *TP53* mutational status (96%) harbored 1 to 4 *TP53* mutations. Among the tested pediatric patients (n = 11/22), 7 (64%) did not harbor somatic *TP53* mutations (BMF, n = 5; MDS not otherwise specified with multilineage dysplasia, n = 1; refractory cytopenia of childhood, n = 1).

**Table 1.** **Patient demographics and transplant procedure**

Variable	All, N = 45 (22 pediatric patients)		Non-HM, n = 20 (13 pediatric patients)	HM, n = 25 (9 pediatric patients)	Missing data, n (%)	*P* value
**Year of first HSCT**					0	.914
Median (IQR)	2021 (2018-2023)		2021 (2018-2023)	2023 (2020-2023)		
**Age at HSCT**					0	.015
Median (IQR), y	18 (13-35)		15 (11-29)	25 (16-38)		
**Sex**	n	%	n (pediatric patients)	n (pediatric patients)	0	1.000
Female	22	49	8 (4)	14 (5)		
Male	23	51	12 (9)	11 (4)		
**Ethnicity**	n	%	n (pediatric patients)	n (pediatric patients)	0	
Arabic	7	16	5 (5)	2 (2)		
East African	2	4	1 (0)	1 (0)		
Finnish	17	38	7 (4)	10 (2)		
Latino	1	2	0	1 (0)		
North African	3	7	0	3 (2)		
White	15	33	7 (4)	8 (3)		
***ERCC6L2* genotype**	n	%	n (pediatric patients)	n (pediatric patients)	0	
Homozygous	30	67	13 (8)	17 (8)		
Compound heterozygous	15	33	7 (5)	8 (1)		
**Indication for first HSCT**	n (pediatric patients)	%			0	
BMF, transfusion-dependent	12 (11)	27				
Proactive for progressive ED	8 (2)	18				
MDS, hypoplastic (*TP53-*mutation status NA)	1 (1)	2				
MDS NOS with single lineage dysplasia∗	2 (2)	4				
MDS NOS with multilineage dysplasia†	5 (2)	11				
MDS; RCC	1 (1)	2				
MDS with mutated *TP53*‡	6 (0)	13				
MDS/AML with mutated *TP53*	3 (2)	7				
MDS/AML with myelodysplasia-related cytogenetic abnormality (*TP53*-mutation status NA)	1 (0)	2				
AML with mutated *TP53*	5 (0)	11				
Other HM (ALL)	1 (1)	2				
***TP53-*mutated clone(s) at HSCT**	n (pediatric patients)	%	n (pediatric patients)	n (pediatric patients)	12 (27)	
Yes	26 (4)	79	8 (0)	18 (4)		
No	7 (7)	21	5 (5)	2 (2)		
**Karnofsky/Lansky performance score at HSCT**	n (pediatric patients)	%	n (pediatric patients)	n (pediatric patients)	5 (11)	
90-100	33 (17)	83	16 (9)	17 (8)		
70-80	7 (2)	17	1 (1)	6 (1)		
**The time between first hematological presentation and HSCT**					0	
Median (IQR), mo	18 (7-53)		26 (6-54)	12 (7-38)		.664
**Donor (HLA match)**	n (pediatric patients)	%	n (pediatric patients)	n (pediatric patients)	0	
Matched sibling donor	7 (3)	15	3 (2)	4 (1)		
Matched unrelated donor (≥10/10)	28§ (11)	63	10 (5)	18§ (6§)		
Mismatched unrelated donor (9/10)	4 (4)	7	3 (3)	1 (1)		
Mismatched unrelated donor (<8/8)	1 (1)	2	0	1 (1)		
Haploidentical donor	4 (4)	9	3 (3)	1 (1)		
Umbilical cord blood donor	2 (0)	4	1 (0)	1 (0)		
**Stem cell source**	n (pediatric patients)	%	n (pediatric patients)	n (pediatric patients)	2 (4)	
BM	20§ (17)	45	12 (11)	8§ (6§)		
PBSC	22 (6)	50	7 (2)	15 (4)		
CB	2 (0)	5	1 (0)	1 (0)		
**Conditioning intensity and regimen**	n (pediatric patients)	%	n (pediatric patients)	n (pediatric patients)	0	
**MAC, total**	27§ (11§)	59	7 (3)	20§ (8§)		
Flu/Treo14 ± TT	19 (6)	41	7 (3)	12 (3)		
Bu/Cy ± Mel	3 (2)	7	0	3 (2)		
Bu/Flu ± TT	5§ (3§)	11	0	5§ (3§)		
**RIC/non-MAC, total**	19 (12)	41	13 (10)	6 (2)		
Flu/Cy ± TBI 2 cGy	13 (9)	28	10 (8)	3 (1)		
Flu/Mel	1 (0)	2	0	1 (0)		
Bu/Flu ± Cy	4 (3)	9	3 (2)	1 (1)		
Bu/Flu/Ven	1 (0)	2	0	1 (0)		
**In vivo T-cell depletion**	n (pediatric patients)	%	n (pediatric patients)	n (pediatric patients)	2 (4)	
ALEM	4 (2)	9	2 (2)	2 (0)		
Rabbit-ATG	35§ (19)	88	17 (11)	18§ (8§)		
None	5 (2)	11	1 (0)	4 (2)		
**Nucleated cell dose**					9 (20)	.181
10^8^/kg, median^30^	6.60 (0.15-29.24)		6.30 (0.45-10.10)	7.80 (0.15-29.24)		
**CD34^+^ cell dose**					8 (19)	.484
10^6^/kg, median^30^	7.60 (1.20-15.84)		7.74 (1.95-15.84)	5.68 (1.20-13.37)		
**GVHD prophylaxis**			n (pediatric patients)	n (pediatric patients)	2 (4)	
CsA ± MMF/MTX	31§ (15§)	70	14 (9)	17§ (6§)		
Pt-Cy ± MMF/CsA ± MTX/SLM/TAC	7 (5)	16	4 (3)	3 (2)		
CsA/TAC + MTX ± RIX/ABC	3 (2)	7	1 (1)	2 (1)		
CsA only	3 (1)	7	1 (0)	2 (1)		

ABC, abatacept; ALEM, alemtuzumab; ALL, acute lymphoblastic leukemia; ATG, antithymocyte globulin; Bu, busulfan; CB, cord blood; CsA, cyclosporine; Cy, cyclophosphamide; Flu, fludarabine; Mel, melphalan; MMF, mycophenolate mofetil; MTX, methotrexate; NA, not available; NOS, not otherwise specified; PBSC, peripheral blood stem cells; Pt-Cy, posttransplant cyclophosphamide; RCC, refractory cytopenia of childhood; RIX, rituximab; SLM, sirolimus; TAC, tacrolimus; Treo, treosulfan; TT, thiotepa; Ven, venetoclax.

∗ Including 2 with *TP53*-mutated clones insufficient to meet criteria for multihit *TP53.*

† Including 3 with single *TP53*-mutated clone.

‡ Including 2 with fibrosis.

§ One parameter of the total is related to a second HSCT in a single patient.

One patient underwent 2 HSCTs, and initially received a transplant for transfusion-dependent BMF and received reduced-intensity conditioning (RIC) with fludarabine-cyclophosphamide and a BM graft from a matched sibling donor who tested negative for *ERCC6L2* variants. At 8 months after HSCT, the patient developed MDS and experienced a decline in donor chimerism from 100% to 47%. The patient proceeded to a second HSCT with MAC containing busulfan-fludarabine-thiotepa and a BM graft from a matched unrelated donor 9 months after the first HSCT.^17^

### Characteristics related to the (first) HSCT

Patients underwent the first HSCT from matched sibling donor (n = 7), haploidentical donor (n = 4), matched unrelated donor (HLA ≥10/10, n = 27), mismatched unrelated donor (HLA 9/10, n = 4 or HLA 7/8, n = 1), or umbilical cord blood donor (n = 2). All of the tested related donors (n = 5) were negative for variants in *ERCC6L2*.

Eleven patients with transfusion-dependent BMF received RIC and 1 treosulfan-based MAC. Six patients with proactive HSCT had treosulfan-based MAC, 1 cyclophosphamide-based RIC, and 1 busulfan-based RIC. Six patients with HM received RIC, and the remaining were treated with either treosulfan-based (n = 12) or busulfan-based (n = 7) MAC.

GVHD prophylaxis consisted of cyclosporine base for the majority (n = 36), most often with a combination of methotrexate and/or mycophenolate mofetil (n = 31). Seven patients received posttransplant cyclophosphamide dose (2 doses of 50 mg/kg, n = 4; 2 doses of 25 mg/kg, n = 1; dosing missing, n = 2). In vivo T-cell depletion included antithymocyte globulin (n = 35; 88%) or alemtuzumab (n = 4; 9%).

### Engraftment and GVHD

All patients experienced primary engraftment with the rates shown in Table 2. Two patients developed secondary graft failure (CIn, 4.8%; 95% confidence interval [CI], 0-11.3).

**Table 2.** **HSCT outcomes (first HSCT)**

Variable/condition at transplant	Total, N = 45	Non-HM, n = 20	HM, n = 25	Missing data, n (%)	*P* value
**Time to neutrophil recovery**				3 (7)	.325
Median, after HSCT (IQR), d	17 (13-21)	20 (14-23)	16 (13-19)		
**Time to platelet recovery**				8 (18)	.542
Median, after HSCT (IQR), d	17 (13-23)	18 (13-23)	16 (13-23)		
**Graft failure**				0	1.000
Primary, n (%)	0	0	0		
Secondary, n (%)	2 (4)	1 (5)	1 (4)		
**Donor chimerism at day +100 (± 14 days) after HSCT**				9 (20)	.640
Median, %^30^	100 (86-100)	100 (86-100)	100 (96-100)		
**Follow-up time after HSCT, mo**				0	.285
Median (IQR)	14 (6-35)	21 (7-53)	13 (5-29)		
**Final outcome, n (%)**				0	.056
Alive in remission	30 (66)	16 (80)	13 (50)		
Deceased due to underlying disease	8 (18)	1 (5)	7 (24)		
Deceased due to NRM	7 (16)	3 (15)	4 (15)		

**Figure 2.**
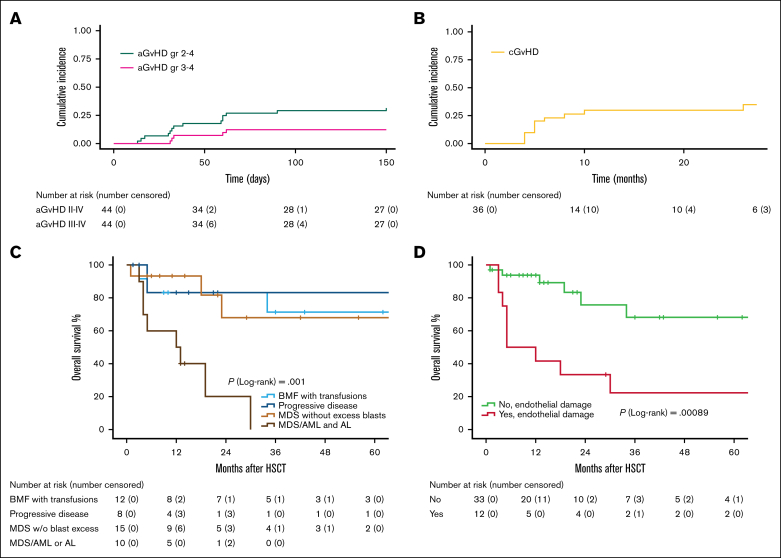
**Cumulative incidence of GVHD and overall survival in 45 subjects with ERCC6L2 disease undergoing HSCT.** CIn of aGVHD and cGVHD (A-B), and OS of 45 subjects with ED undergoing HSCT (C-D). aGVHD includes grades 2 to 4, and 3 to 4, respectively (A), and cGVHD mild and moderate cases (no extensive events recorded). OS stratified by transplant indication (C), and grade 3 to 5 endothelial toxicity events (D).

The CIn of grade 2 to 4, and grade 3 to 4 aGVHD at 100 days were 29% (95% CI, 22-36) and 13% (95% CI, 7-18), respectively. The CIn of cGVHD at 3 years was 34% (95% CI, 26-42; limited in 77%, and moderate in 23% of patients, and no severe events; Figure 2A-B).

### Infections and TRT (grade 3-5)

**Figure 3.**
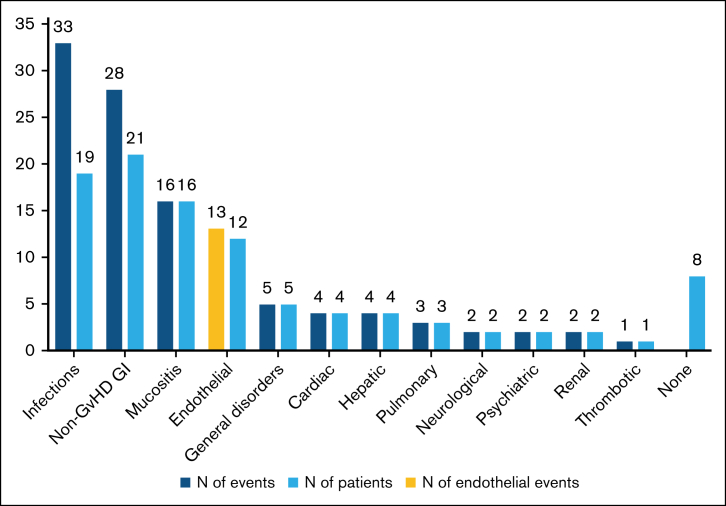
**Number and distribution of nonhematological SAE after HCST recorded in patients with ED.** A detailed list of the events is presented in supplemental Table 3. GI, gastrointestinal.

Detailed data on grade 3 to 5 severe adverse events (SAE) after HSCT were available for 96% of the patients (n = 43), of whom 35 (81%) experienced ≥1 severe complication (Figure 3; supplemental Table 3). However, 5 of the 8 patients without severe TRT suffered from GVHD. The most frequently observed problems were gastrointestinal events unrelated to GVHD (n = 21), infections (n = 19), and mucositis (n = 16). Nine patients had multiple infections. Cytomegalovirus and Epstein-Barr virus (EBV) reactivation were the 2 most frequent pathogens, affecting 6 (14%) and 8 (19%) patients, respectively, with 1 patient who was EBV positive progressing to posttransplant lymphoproliferative disease (PTLD). We did not record disseminated EBV or cytomegalovirus cases. Additionally, 1 patient had fatal mesenteric ischemia unrelated to either GVHD or TA-TMA. We recorded 5 general disorders, including 2 multiorgan failures,^31^ 1 grade 3 hyponatremia, 1 grade 3 weight gain during the first year after transplant, and 1 presumed hemophagocytic lymphohistiocytosis (HLH) diagnosed clinically on day +17 after HSCT (prolonged fever, elevated ferritin and triglyceride, profound cytopenia after engraftment, and atypical histiocytes in histological sample, insufficient to meet HLH-2004 criteria). Most of the 113 recorded SAE occurred early, before day +100 after HSCT, with only 9 complications occurring later: acute respiratory distress syndrome with multiple organ failure, acute respiratory distress syndrome with sepsis, central vein thrombosis, depression, elevated transaminase levels, PTLD, weight gain, and 2 incidences of TA-TMA.

Seven patients underwent a single fraction of total body irradiation (TBI) maximum of 2 Gy in combination with fludarabine-cyclophosphamide RIC without experiencing toxicity. Interestingly, a patient who had localized radiotherapy (20 × 2 cGy) for PTLD developed grade 4 radiodermatitis and mucositis with trismus.

Importantly, a total of 12 of 45 patients (27%) experienced the following grade 3 to 5 complications related to endothelial toxicity: capillary leak syndrome (n = 1), cytokine release syndrome (n = 1), ES (n = 1), PERDS (n = 1), TA-TMA (n = 4), and SOS (n = 4; supplemental Table 3). One patient developed both TA-TMA and SOS. Patients with endothelial damage had various *ERCC6L2* genotypes, but the sample size was insufficient for further comparison. We did not detect statistically significant differences in patient or transplant characteristics between the patients with and without endothelial damage, except for conditioning intensity and outcomes (supplemental Table 4). Nontreosulfan-based MAC was associated with an increased CIn of endothelial complications with an HR of 4.9 (95% CI, 1.1-22.0) compared with RIC. All 4 patients who developed endothelial toxicity after nontreosulfan-based MAC received double alkylator regimens. Treosulfan-based MAC and RIC showed similar outcomes. The median survival time for patients with endotheliopathy was 5 months (95% CI, 0-11). Additionally, the OS was significantly inferior, with 1- and 3-year rates of 42% (95% CI, 14-70) and 22% (95% CI, 0-48), respectively, compared with 94% (95% CI, 85-100) and 68% (95% CI, 45-91; *P* < .001) for those without endothelial complications. Furthermore, patients with endotheliopathy had significantly higher NRM rates than patients without (42% vs 6%; *P* = .010), whereas the incidence of relapse-related deaths was similar (*P* = .181). Endothelial damage events typically occurred early after transplant, with a median onset of 14 days after HSCT (interquartile range [IQR], 6-75; Figure 1). One patient developed TA-TMA at 11 months after HSCT shortly after receiving a CD34^+^-selected stem cell boost for poor graft function.

### Transplant outcome and post-HSCT treatments

With the median follow-up time of 14 months (IQR, 6-35), the 1- and 3-year OSs for all study subjects were 79% (95% CI, 66-91) and 54% (95% CI, 35-73), respectively (supplemental Figure 1A). The 3-year OSs for pediatric patients were 66% (95% CI, 40-92) and for adults 43% (95% CI, 16-70; *P* = .259). The donor or graft type did not significantly affect HSCT outcomes; however, the female sex was associated with inferior OS (HR, 5.5; 95% CI, 1.1-27.0; *P* = .035; supplemental Tables 5-7). Baseline characteristics were generally balanced between female and male subjects with ED. A modest difference in NMR was observed, with females experiencing higher rates than males (*P* = .047; supplemental Table 7). Patients with progressive ED (88% *TP53*-mutated) had a survival probability comparable to patients with transfusion-dependent BMF (20% *TP53*-mutated; Figure 2C). The 1-year OS rate was 83% for both groups (95% CIs, 53-100 and 62-100, respectively), and at 3 years was 83% (95% CI, 53-100) and 71% (95% CI, 43-100), respectively (*P* = .734). Furthermore, the patients with MDS without excess blasts (83% *TP53-*mutated) shared a similar OS, with rates for 1 and 3 years of 93% (95% CI, 81-100) and 68% (95% CI, 37-100), respectively. In contrast, patients with MDS/AML or acute leukemia (eg, history of excess blasts ≥10% at any time before HSCT, 100% *TP53*-mutated) had a median survival time of 12 months (95% CI, 0-24; HR, 6.1; 95% CI, 1.6-24.3; *P* < .001). Overall, 14 of 25 (56%) patients with HM were alive at the end of follow-up, but survival was notably poorer among patients with ≥10% blast excess, with only 2 out of 10 patients alive. One patient with AML achieved complete remission with minimal residual disease (MRD) negativity at the end of a follow-up of +14 months after HSCT, after receiving prophylactic post-HSCT treatment with decitabine and venetoclax. The single patient who received translpant due to acute lymphoblastic leukemia was also in CR at the +13-month follow-up after HSCT.

In addition, 2 patients were treated with post-HSCT therapies (supplemental Table 1). One patient with MDS/AML received post-HSCT hypomethylating agent (HMA), initiated at 10 weeks, and 4 donor lymphocyte infusions due to MRD positivity. The patient achieved MRD negativity at +18 months, but at +26 months experienced morphological relapse. A combination of HMA and venetoclax did not elicit a response, and the patient succumbed. One patient, who receive a transplant due to MDS with *TP53* mutations and without excess blasts, had an MRD relapse 12 months after HSCT. The patient first received HMA without a response, and at 18 months a combination of HMA and venetoclax was started. The patient achieved MRD negativity and was alive at the end of the follow-up of 22 months.

We observed 15 deaths at a median of 5 months (IQR, 4-19) following HSCT: 7 due to NRM and 8 due to relapse. Estimated EFS rates at 1 and 3 years were 77% (95% CI, 64-89) and 57% (95% CI, 39-75), respectively (supplemental Figure 1B). The CIn of NRM at 3 years was 19% (95% CI, 12-26). Causes of NRM included GVHD (n = 1), organ damage (n = 5, of which 4 involved endothelial toxicity), and PTLD (n = 1). Due to the limited number of events, the multivariable Cox regression model was restricted to covariates selected based on univariable significance (supplemental Table 5). In the multivariable analysis, female sex and a history of excess blasts before HSCT were independently associated with poorer OS, with HRs of 3.8 (95% CI, 1.2-12.2; *P* = .027) and 5.7 (95% CI, 1.6-19.9; *P* = .007), respectively (supplemental Table 6). Nontreosulfan-based MAC showed a trend toward increased mortality compared with RIC (HR, 4.0; 95% CI, 1.0-16.2; *P* = .054).

The CIn of morphological relapses at 3 years was 24% (95% CI, 16-32). Seven out of the 8 relapsed patients had a history of excess blasts before HSCT. The median onset for morphological relapse was 8 months (IQR, 3-18), and all died within the median of 13 months (IQR, 4-28). The DFSs for 1 and 3 years were 87% (95% CI, 77-98) and 73% (95% CI, 56-91), respectively (supplemental Figure 1C).

## Discussion

**Figure 4.**
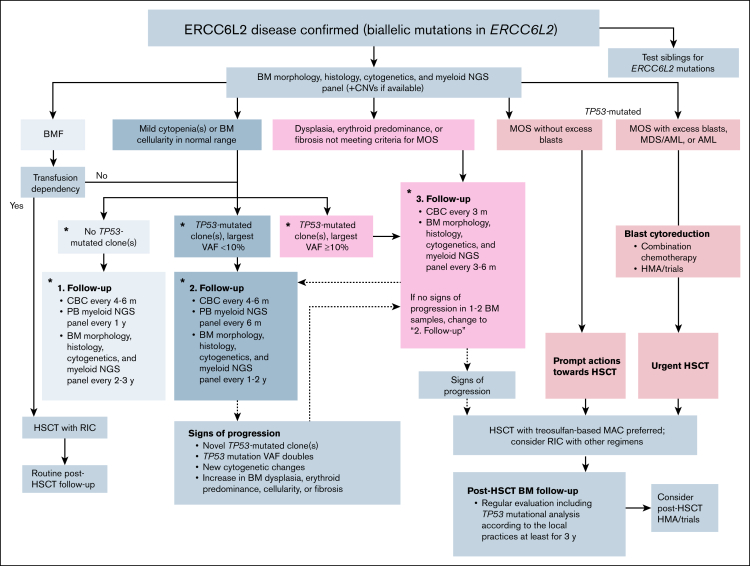
**Approach to initial investigations, follow-up, and treatment of patients with ED.** The pre-HSCT follow-up and workup for HSCT depends on the current findings in the BM and *TP53* mutational status in patients without HM, and are based on expert opinion. We suggest screening for myeloid NGS panel to identify possible co-occurring driver mutations in addition to *TP53*. ∗Indicates expert opinion. CBC, complete blood count; CNV, copy number variation; NGS, next-generation sequencing; PB, peripheral blood; VAF, variant allele frequency.

Our study represents the first multicenter analysis of HSCT for ED, providing valuable insights into transplant characteristics, outcomes, and TRTs associated with the life-threatening IBMFS. We gathered comprehensive data, including a detailed questionnaire of post-HSCT TRTs from each study subject (supplemental Table 1). These exceptionally complete data together with our clinical experience enabled us to outline follow-up and treatment strategies tailored to each stage of the ED continuum (Figure 4). Each patient requires a comprehensive diagnostic workup at initial presentation, including BM aspirate for morphological assessment, cytogenetics, myeloid mutation next-generation sequencing panel, and copy number variation analysis for *TP53*-mutated clones when available. Additionally, a trephine biopsy is needed for histological analysis, and a more accurate examination of cellularity, dysplasia, and fibrosis. Based on the previously recognized increased risk for developing HMs, we suggest a variant allele frequency cutoff of 10% for the largest *TP53-*mutated clone as an indication for more frequent BM surveillance as an expert opinion.^32^ Although very rare, additional driver mutations may occur alongside *TP53* mutations (Kilpivaara O and Wartiovaara-Kautto U, unpublished data, December 2025; Hakkarainen et al^8^); hence, we recommend using a myeloid mutation next-generation sequencing panel instead of *TP53* sequencing alone for surveillance.

Genetic screening of related donors is a critical consideration in IBMFS; however, only 46% of the related donors in this study were tested for *ERCC6L2*. Here, none was identified as heterozygous carriers. Although healthy heterozygous carriers are considered suitable donors in recessive disorders such as Fanconi anemia and Shwachman-Diamond syndrome,^33^, ^34^, ^35^ their use in ED requires caution due to a lack of supporting data. Even though the number of haploidentical donors in our study was limited, the results are promising in other IBMFSs with more data.^12^^,^^36^

Among our key findings in ED is the elevated frequency of grade 3 to 5 endothelial toxicity in 27% of both pediatric and adult patients. For comparison, a recent study reported a 15.6% incidence of vascular toxicity among pediatric HSCTs (the median age of 7 years vs 13 years among our pediatric patients).^37^ In the adult recipients of HSCT, references for endothelial toxicity incidence stem from studies evaluating each endothelial complication individually. Moreover, clinical manifestations often overlap between different syndromes, the grades of SAE are not consistently reported, and several proposed consensus criteria exist for TA-TMA. Thus, the reported incidences vary significantly, and are ∼3% to 12% for TA-TMA; 10% to 15% for SOS (with recent estimates <5%); 13% for ES; and <5% for PERDS.^29^^,^^38^, ^39^, ^40^, ^41^, ^42^ In addition to the 12 patients with endothelial toxicity in this study, 1 patient developed a cascade presumed as HLH after engraftment, in which the pathophysiology is most likely connected to endothelial damage.

Similar to previous reports, we found endothelial damage to be associated with inferior OS,^37^^,^^42^, ^43^, ^44^ with a higher risk in patients receiving nontreosulfan-based MAC/double alkylator regimens compared with RIC. However, we observed endothelial complications also in patients who received RIC. We hypothesize that DNA damage induced by alkylating agents, combined with the involvement of ERCC6L2 in DNA repair, may contribute to the increased susceptibility to endothelial toxicity in ED. This parallels the heightened sensitivity to alkylating agents observed in Fanconi anemia cells in vitro.^45^ In our series, none received a myeloablative TBI-containing regimen, and 7 conditioned with RIC including low-dose TBI (2 Gy) without experiencing any radiation-related toxicity. However, we report a severe toxicity related to localized radiotherapy supporting our previous observations.^8^ Although, not recorded in our series, we recommend avoiding TBI-based MAC in patients with ED based on this and previous observations, and the observed radiosensitivity in DNA repair disorders.

The rates of GVHD were not unusually high and the patients with endothelial complications did not have more GVHD than the patients without. Overall, the frequency and distribution of grade 3 to 5 infections and organ-specific adverse events, other than endothelial complications, were consistent with general expectations in HSCT. However, the CIn of NRM (19%) appears high compared with general HSCT outcomes, with reported incidences of 9.5% and 11%.^46^^,^^47^ Nonetheless, this includes only early events after HSCT due to the limited follow-up time in our study. Increased susceptibility to NRM and organ failure is observed also in other IBMFS.^13^^,^^14^^,^^33^

Although specific supportive care measures are not established, our data suggest conditioning should be individually tailored to minimize toxicity (Figure 4). In patients dependent on transfusion, RIC leads to favorable outcomes. For those showing early signs of progressive ED, prompt consideration of proactive HSCT is crucial: the disease can accelerate to overt HM within months. However, our data here, and in previous studies, as well as clinical experience suggest that disease progression can be detected through careful and repeated monitoring.^8^ Patients at high risk of malignant transformation typically show accumulation or expanding BM *TP53-*mutated clones, increasing dysplasia, cellularity, reticulin fibrosis, or erythroid predominance, all of which can be identified through clinical and laboratory screening. It is important to note that complete blood count follow-up alone is insufficient for this purpose,^8^ and that risk for progression associated with *TP53*-mutated clones vary individually. A “watch-and-wait” approach is feasible for patients without HM who can comply with close follow-ups, particularly in childhood and adolescence, given the median onset age of 37 years for HM.^8^ Moreover, our data demonstrate that the OS is comparable among patients with BMF, progressive ED, and MDS if without excess blasts. Monitoring and intervention decisions should be individualized and discussed in a multidisciplinary team, incorporating both laboratory and clinical findings, with shared decision-making involving patients and their families.

In this study, patients with a history of excess blasts ≥10% had a particularly poor outcome, reinforcing the need for transplant before disease progression to overt malignancy. This finding aligns with studies on other IBMFSs, where HSCT performed before excess blasts or leukemic transformation results in better outcomes.^33^^,^^48^^,^^49^ Here, it is also important to recognize that patients with ED often develop chemoresistance, which may prevent some from proceeding to HSCT.^8^ Further studies are needed to include patients with a history of blast counts of 5% to 9%, as this group was not represented among our study subjects. In addition to the BM blast count, female sex associated with worse OS. Our findings suggest that the inferior OS in female recipients is mainly driven by higher NRM rather than relapse. This phenomenon is intriguing and calls for further studies as it differs somewhat from previous studies linking male sex to inferior OS after allogeneic HSCT,^50^^,^^51^ but should be interpreted with caution given the limited number of events in this study.

For patients with HM, individualized risk assessment is vital, given the poor prognosis of *TP53*-mutated disease and uncertainty regarding the benefit of conditioning intensity.^52^ Although our study did not identify significant predictors of endothelial damage based on patient or HSCT characteristics, other than nontreosulfan-based MAC, various contributing factors have been reported.^53^ Treosulfan-based MAC, with a less intense alkylating profile than busulfan, may offer potential benefits in reducing toxicity. However, we recognize that additional contributing factors exist, as the study series is heterogeneous and limited in size.

Despite the restrictions in the number of patients, lack of longitudinal data, and retrospective design, our study benefits from the strengths of a collaborative, multicenter, international approach, particularly given the increasing recognition of ED in recent years. Furthermore, the novelty of ED and the occurrence of early events constrain the study’s follow-up. However, the median follow-up time of 14 months captures the main points of interest, endothelial toxicity events and relapses, occurring at a median of 14 days and 8 months, respectively. Longer observation is, however, recommended, particularly to record the potential development of secondary cancers. We suggest long-term monitoring of patients with ED in centers with hematological expertise. For all patients who received transplants with *TP53*-mutated clone(s), we propose regular BM evaluation including *TP53* mutational analysis performed according to local practices for ≥3 years after HSCT as an expert opinion. Novel approaches in the treatment of *TP53*-mutated HMs, including prophylactic and active post-HSCT therapies, are important for patients with ED. Here, the longest DFS among the patients with AML was observed at last control at 14 months in a patient who received post-HSCT treatment of preemptive HMA and venetoclax after successful HSCT with venetoclax combined with fludarabine/busulfan RIC.^54^

In conclusion, recessively inherited ED predisposes patients to an aggressive HM, with timely HSCT currently being the only treatment with the potential to save lives. Nonetheless, patients remain at high risk for endothelial damage and relapse after HSCT. Our findings support the use of HSCT in ED in its early stages, before transformation to a highly aggressive malignancy with blast excess, to improve outcomes. The increased incidence of endothelial toxicity in patients with ED highlights the critical need for careful conditioning regimen selection and vigilance during the HSCT course and the following months. RIC appears to reduce the risk of endothelial complications, whereas for those requiring MAC, treosulfan-based MAC is preferable for safety. Future research and international collaboration, along with long-term follow-up, are essential for further understanding of ED and improving patient outcomes.

Conflict-of-interest disclosure: The authors declare no competing financial interests.
