# Supplementary material for: Allogeneic hematopoietic stem cell transplantation in ERCC6L2 disease
Source: Blood Adv. 2026 Feb 4;10(7):2525–37. doi: 10.1182/bloodadvances.2025018349 (PMC13083646; doi:10.1182/bloodadvances.2025018349)
Supplement: Supplemental Tables and Figure [file BLOODA_ADV-2025-018349-mmc1.pdf]

Supplemental material

Supplemental Table 1. Detailed study subject and transplant characteristics (see Excel table).

Supplemental Table 2. The evaluation of the impact of the transplantation era.

Supplemental Table 3. Detailed distribution of adverse events grades 3-5 post-transplant graded according to CTCAE 5.0.

Supplemental Table 4. Characteristics and comparison of the patients with endothelial damage (n = 12) vs. those without (n = 33 for patients, n=34 for transplants).

Supplemental Table 5. Univariable analysis of OS for all study subjects stratified by baseline characteristics.

Supplemental Table 6. Multivariable Cox regression model of OS stratified by covariates selected based on univariate significance.

Supplemental Table 7. The evaluation of the impact of female sex.

Supplemental Figure 1. A. The overall survival probability of all study subjects. B. Disease-free survival for all study subjects. C. Event-free survival for all study subjects.

Supplemental Table 2. The evaluation of the impact of the transplantation era.

We divided the years of the transplantations into three different eras for comparison to evaluate the possible effect of the transplant year. We grouped transplants into three periods: 1. Early (2004-2015), n=7; 2. Middle (2017-2020), n=12, 3. Recent (2021-2024), n=26.

Variable	Early, 2004-15, n=7	Middle, 2016-20, n=12	Recent, 2021-24, n=26	*P*
**Patient characteristics**				
Pediatric patients ≤ 18 years, n (%)	3 (43)	6 (50)	13 (50)	1.000
Female sex, n (%)	3 (43)	8 (67)	11 (42)	0.387
TP53 mutated cases / N of analyzed cases	3/3, data missing, n=4 (57%)	7/8; data missing, n= 4 (33 %)	16/22, data missing n=4 (15%)	0.536
**Indication for HSCT**				0.723
BMF with transfusion dependent, n (%)	3 (43)	3 (25)	6 (23)	
Proactive for progressive ED, n (%)	0	2 (17)	6 (23)	
HM, n (%)	4 (57)	7 (58)	14 (54)	
**Conditioning intensity**				0.594
RIC, n (%)	4 (57)	3 (25)	12 (46)	
Treosulfan-based MAC, n (%)	2 (29)	6 (50)	11 (42)	
Non-treosulfan-based MAC, n (%)	1 (14)	3 (25)	3 (12)	
**Outcome**				
1-year OS, % (95% CI)	71 (38-1)	67 (40-93)	88 (75-1)	0.196
3-year OS, % (95% CI)	43 (62-80)	42 (14-70)	66 (27-1)	0.196
Endothelial toxicity events, n (%)	3 (43)	5 (42)	4 (15)	0.101
NRM, n (%)	1 (14)	4 (33)	2 (8)	0.119
Relapse, n (%)	3 (43)	3 (25)	2 (8)	0.072
Median follow-up time, months (IQR)	19 (13-109)	28 (5-64)	13 (6-22)	0.640 (Kruskal-Wallis)

Abbreviations: AML, acute myeloid leukemia; BMF, bone marrow failure; ED, ERCC6L2 disease; HM, hematological malignancy; IQR, interquartile range; MAC, myeloablative conditioning; MDS, myelodysplastic syndrome; NRM, non-relapse mortality; OS, overall survival; RIC, reduced intensity conditioning.

Supplemental Table 3. Detailed distribution of adverse events grades 3-5 post-transplant according to CTCAE 5.0.

Type of severe adverse event	N of events	N of patients (%)
**Infectious events;** *data missing, n = 2 (4%)*	**33**	**19 (48)**
Bacterial	12	
Fungal	1	
Viral	20	
**Type of viral infections**		
Cytomegalovirus reactivation	6	
Epstein-Barr virus reactivation	8	
Other*	5	
Post-transplant lymphoproliferative disease	1	
**Cardiac;** *data missing, n = 2 (4%)*	**4**	**4 (9)**
Hypertension; hypotension	3; 1	
**Endothelial complications,** *no missing data*	**13†**	**12 (27)**
Capillary leak syndrome	2	
Cytokine release syndrome	1	
Engraftment syndrome	1	
Peri-engraftment respiratory distress syndrome	1	
Transplant-associated thrombotic microangiopathy	4	
Veno-occlusive disease	4	
**Gastrointestinal (not related to GvHD);** *data missing, n = 2 (4%)*	**28**	**21 (47)**
Nausea/vomiting	10	
Diarrhea	2	
Feeding problems/anorexia	14	
Bleeding	1	
Visceral arterial ischemia	1	
**General disorders;** *data missing, n = 2 (4%)*	**5**	**5 (11)**
Multiorgan failure	2	
Hemophagocytic lymphohistiocytosis	1	
Hyponatremia	1	
Weight gain	1	
**Hepatic (not related to SOS);** *data missing, n = 2 (4%)*	**4**	**4 (9)**
Increase in transaminase levels (ALAT or ASAT) or alkaline phosphatase (AFOS)	4	
**Neurological;** *data missing, n = 2 (4%)*	**2**	**2 (4)**
Ataxia	1	
Seizures	1	
**Oral;** *data missing, n = 2 (4%)*	**16**	**16 (36)**
Mucositis	16	
**Psychiatric;** *data missing, n = 2 (4%)*	**2**	**2 (4)**
Anxiety	1	
Depression	1	
**Pulmonary;** *data missing, n = 2 (4%)*	**3**	**3 (6)**
Acute respiratory distress syndrome	3 ‡	
**Renal (not related to TMA or MOF);** *data missing, n = 2 (4%)*	**2**	**2 (4)**
Acute kidney injury	2	
**Thromboembolic events;** *data missing, n = 2 (4%)*	**1**	**1 (1)**
**No reported toxicities**		**8 (17)**

Some patients experienced multiple events in the same category. *Other viral infection agents, BK polyomavirus, human herpesvirus, adenovirus, and human papillomavirus. †One patient experienced TMA and VOD. ‡One patient experienced additional multiorgan failure (MOF).

Supplemental Table 4. Characteristics and comparison of the patients with endothelial damage (n = 12) vs. those without (n = 33 for patients, n=34 for transplants).

	**Endothelial toxicity, n=12**	**Others, n=33; n for HSCT=34**	
**Variable**	**n (%)**	**n (%)**	***p***
**Age at 1st HSCT**			0.995
Median, years (IQR)	19 (12-40)	18 (14-34)	
**Sex**			0.514
Female/Male, n (%)	7/5 (51/49)	15/16 (45/55)	
**Year of 1st HSCT**			0.135
Median (IQR)	2020 (2016-2022)	2022 (2019-2023)	
**Pre-HSCT treatment (1st HSCT)**			0.362
Combination chemotherapy	4 (33)	5 (15)	
Hypomethylating agent	1 (8)*	1 (3)	
Immunosuppressive therapy	0	4 (12)	
None/transfusions	7 (58)	22 (67)	
**Indication for HSCT**			0.909
BMF with transfusion dependency	3 (25)	9 (27)	
Proactive for progressive ED	2 (17)	8 (24)	
Hematological malignancy	7 (58)	17 (51)†	
**Conditioning intensity**			0.07
Treosulfan-based MAC	5 (42)	14 (41)	HR 1.7 (95% CI 0.4-7.1) compared to RIC
Non-treosulfan-based MAC	4 (33)	4 (12)†	**HR 4.9 (95% CI 1.1-22.0) compared to RIC**
RIC	3 (25)	16 (47)	
**In-vivo T-cell depletion**			0.826
Alemtuzumab	1 (8)	3 (9)	
rATG	9 (75)	26 (76)†	
None	2 (17)	3 (9)	
**Donor type, HLA Match**			0.696
MUD ≥ 10/10	6 (58)	21 (62)†	
MUD 9/10	2 (17)	3 (9)	
MSD	2 (17)	5 (15)	
mMUD <8/8	1 (8)	2 (6)	
HID	1 (8)	3 (9)	
**Graft type**			0.453
BM	7 (58)	13 (38)†	
PBSC	5 (42)	17 (50)	
CB	0	2 (6)	
**Acute GVHD, grade II-IV**			0.284
Yes, n (%)	5 (42)	9 (27)	
**Chronic GVHD**			0.715
Yes, n (%)	3 (27)	11 (33)	
**Outcome**			**0.00089**
Deceased, n (%)	9 (75)	6 (18)	
**Cause of death**			**0.001**
NRM	5 (42)	2 (7)	**0.010**
Relapse	4 (33)	4 (13)	0.181

Due to single non-reported details, numbers may not add to the total or 100 %. * Combined with investigational anti-TIM3 treatment. † Parameter related to one patient's 2nd HSCT. Abbreviations: BM, bone marrow; CB, cord blood; CI, confidence interval; ED, ERCC6L2 disease; GvHD, graft-versus-host-disease; HID, haploidentical donor; HLA, human leukocyte antigen; HR, hazard ratio; HSCT, hematopoietic stem cell transplantation; IQR, interquartile range; MAC, myeloablative conditioning; MSD, matched sibling donor; MUD, matched unrelated donor; mMUD, mismatched unrelated donor; NRM, non-relapse mortality; PBSC, peripheral stem cells; rATG, anti-thymocyte globulin; RIC, reduced intensity conditioning.

Supplemental Table 5. Univariable analysis of OS for all study subjects stratified by baseline characteristics related to the 1st HSCTs.

Variable	3-y OS, % (95% CI)	*P* value	HR (95% CI)
**Year of 1st HSCT**		0.468	
< 2020 (n=16)	50 (26-75)		
≥2020 (n=29)	48 (14-82)		
**Age at HSCT**		0.259	
Pediatric patients ≤ 18 y (n=22)	66 (40-92)		
Adults, >18 y (n=23)	43 (16-70)		
**Sex**		0.035	
Female (n=22)	36 (11-61)		5.5 (1.1-27.0)
Male (n=23)	75 (51-98)		
***TP53* clone(s)**		0.233	
Yes (n=26)	43 (16-69)		
No (n=7)	67 (13-100)		
**Chromosomal changes**		0.002	
Yes, (n=15)	18 (0-47)		5.1 (1.7-15.8)
No (n=25)	70 (46-93)		
**Indication for HSCT**		0.001	
BMF with transfusion dependency (n=12)	71 (43-100)		
Proactive for progressive ED (n=8)	83 (54–100)		
MDS without excess blasts (n=15)	68 (44-92)		
MDS/AML, AL (n=10)	0		6.8 (2.2–20.3)
**Time between the 1st hematological presentation and HSCT**		0.157	
<12 months (n=17)	30 (3-57)		
12-24 months (n=10)	64 (30-98)		
>24 months (n=18)	82(64-100)		
**Donor type**		0.907	
MSD (n=7)	58 (34-83)		
MUD (n=27)	43 (6-80)		
MMUD (n=7)	57 (8-100)		
HID (n=4)	75 (32-100)		
**Graft type**		0.877	
BM (n=19)	51 (25-77)		
PBSC (n=22)	49 (14-83)		
CB (n=2)	50 (0-1)		
**Conditioning intensity**		0.006	
RIC (n=19)	65 (34-95)		
Treosulfan-based MAC (n=19)	55 (26-84)		1.8 (0.4-8.5)
Non-treosulfan-based MAC (n=7)	21 (0-56)		4.9 (0.9-8.5)

Abbreviations: BM, bone marrow; CB, cord blood; CI, confidence interval; ED, ERCC6L2 disease; HID, haploidentical donor; HR, hazard ratio; HSCT, hematopoietic stem cell transplantation; MAC, myeloablative conditioning; MSD, matched sibling donor; MUD, matched unrelated donor; mMUD mismatched unrelated donor; PBSC, peripheral stem cells; RIC, reduced intensity conditioning.

Supplemental Table 6. Multivariable Cox regression model of OS stratified by covariates selected based on univariate significance. The overall model was statistically significant (p < 0.001).

	**OS, 15 deaths**	
**Variable**	**HR (95% CI)**	***P* value**
History of excess blasts before HSCT	5.7 (1.6–19.9)	0.007
Female sex	3.8 (1.2–12.2)	0.027
Conditioning intensity		
Treo-based MAC vs RIC	1.5 (0.4–5.3)	0.557
Non-Treo-based MAC vs RIC	4.4 (0.95–19.9)	0.058

Abbreviations: HSCT, hematopoietic stem cell transplantation; HR, hazard ratio; MAC, myeloablative; RIC, reduced intensity conditioning; Treo, treosulfan.

In the multivariable analysis, a history of excess blasts before HSCT was a stronger and statistically significant predictor of overall survival (OS; HR 4.5, 95% CI 1.2–16.0, p = 0.022) compared to the presence of chromosomal abnormalities, which showed only a borderline association (HR 3.4, 95% CI 0.88–13.5, p = 0.077). Given its stronger and statistically significant effect, excess blasts were selected for inclusion in the final model. Similarly, age (pediatric vs. adult) was not statistically significant (HR 1.7, 95% CI 0.4–6.5, p = 0.445) and was therefore not included in the final model.

Supplemental Table 7. The evaluation of the impact of female sex.

	Female, n=22	Male, n= 23	
**Variable**	**n (%)**	**n (%)**	***p***
**Age at 1st HSCT**			0.791
Median, years (IQR)	22 (13-35)	16 (12-35)	
**Pediatric patients**			0.306
N (%)	9 (41)	13 (57)	
***TP53*-mutated clones**			0.182
Yes, n (%)	18	15	
**Year of 1st HSCT**			0.728
Median (IQR)	2021 (2019-2023)	2022 (2018-2023)	
**Pre-HSCT treatment (1st HSCT)**			1.000
Combination chemotherapy	5 (23)	4 (18)	
Hypomethylating agent	1 (5)	1 (5)	
Immunosuppressive therapy	2 (9)	2 (9)	
None/transfusions	14 (64)	15 (65)	
**Indication for HSCT**			0.370
BMF with transfusion dependency	7	5	
Proactive for progressive ED	3	7	
Hematological malignancy	11	11	
Patients with history of excess blasts	5	5	1.000
**Conditioning intensity**			0.946
Treosulfan-based MAC	9	10	
Non-treosulfan based MAC	3	4	
RIC	10	9	
**In-vivo T-cell depletion**			0.001
Alemtuzumab	1 (5)	1 (5)	
rATG	15 (68)	19 (90)	
None	6 (27)	1 (5)	
**Donor type, HLA Match**			0.344
MUD ≥ 10/10	14 (64)	13 (57)	
MUD ≤ 9/10	2 (9)	5 (22)	
MSD	5 (23)	2 (9)	
HID	1 (5)	3 (13)	
**Sex mismatch between donor/recipient**			0.536
Yes, n (%)	8	11	
**Graft type**			0.630
BM	11	8	
PBSC	10	12	
CB	1	1	
**Acute GVHD, grade 2-4**			0.846
Yes, n (%)	7	7	
**Chronic GVHD**			1.00
Yes, n (%)	7	7	
**Endothelial toxicity event, grade 3-5**			0.141
Yes, n (%)	7	5	
**Cause of death**			**0.0004**
NRM	6	1	**0.0470**
Relapse	5	3	0.4951

Abbreviations explained in the footnote of Supplemental Table 5.

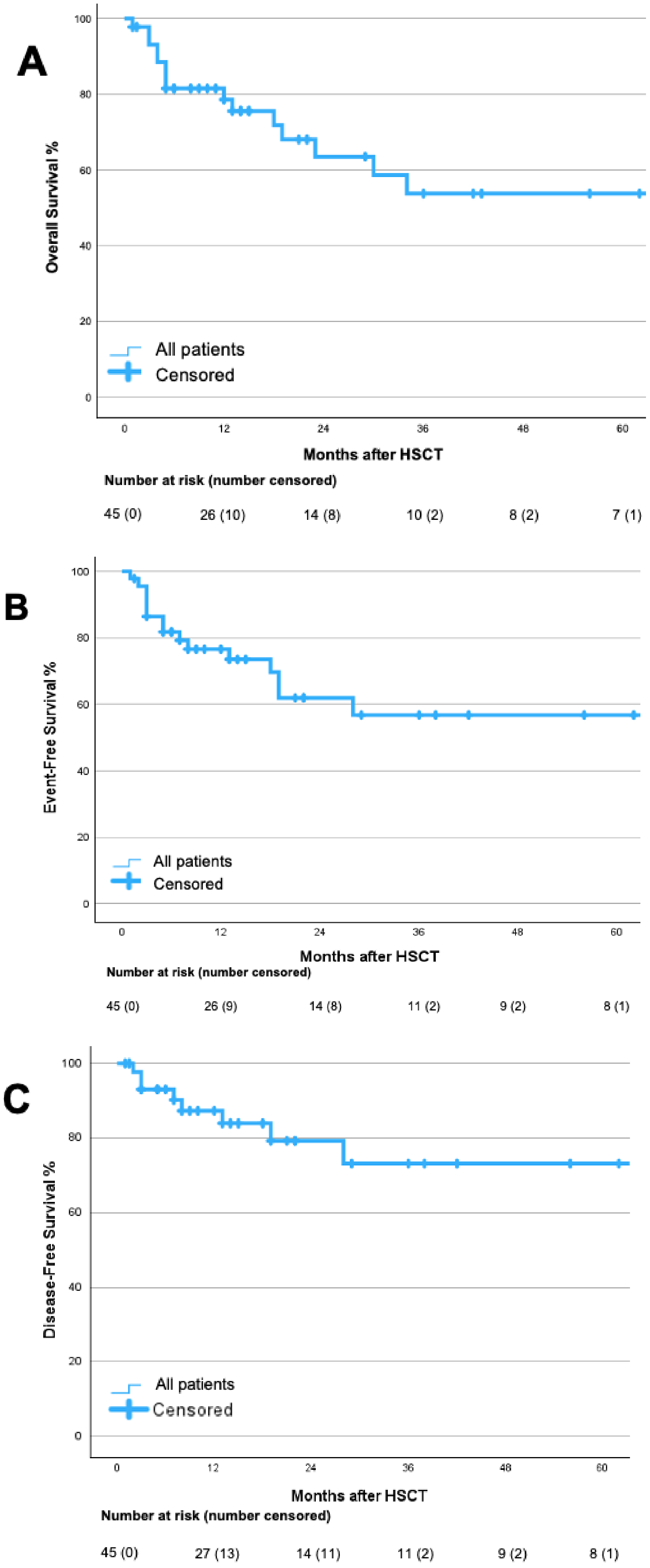

Supplemental Figure 1. A. The overall survival probability of all study subjects. B. Disease-free survival for all study subjects. C. Event-free survival for all study subjects.
